# “C‐reactive protein levels and risk of dementia”: Subgroup analyses in Mendelian randomization are likely to be misleading

**DOI:** 10.1002/alz.12743

**Published:** 2022-08-21

**Authors:** Stephen Burgess

**Affiliations:** ^1^ MRC Biostatistics Unit University of Cambridge Cambridge UK; ^2^ Cardiovascular Epidemiology Unit Department of Public Health and Primary Care University of Cambridge Cambridge UK

Letter in response to “C‐reactive protein levels and risk of dementia—Observational and genetic studies of 111,242 individuals from the general population” by Hegazy and colleagues.

Mendelian randomization is the use of genetic variants analogously to randomized treatment allocation in a trial to make causal inferences from observational data. In their recent article, Hegazy and colleagues assessed associations between genetic variants in the *CRP* gene region—which they treated analogously to random allocation to increased levels of C‐reactive protein (CRP)—and risk of dementia.^1^ However, rather than assessing associations in the population as a whole, they assessed associations in strata of the population defined according to body mass index (BMI): those with BMI ≤25 kg/m^2^, and those with BMI >25 kg/m^2^. They observed positive associations between variants and dementia risk in the low BMI stratum, and inverse associations (although mostly not statistically significant) in the high BMI stratum. The authors interpreted the positive associations as evidence that CRP has a deleterious effect on dementia risk in low BMI individuals. However, there are reasons to be skeptical of this interpretation.

The genetic “randomization” exploited in Mendelian randomization occurs at conception. Therefore, all subgroup analyses in a Mendelian randomization investigation are improper subgroup analyses, as stratification is always based on a post‐randomization covariate—that is, a variable measured after randomization.^2^ The only exceptions are subgroup analyses based on variables that logically cannot be caused by the genetic variants, such as age, sex, and ancestry.

When stratification in a randomized trial is based on a post‐randomization covariate, exchangeability is not guaranteed for the stratified trial arms; in other words, randomization is broken within the strata. This can be understood as an example of collider bias: stratifying (or equivalently, conditioning or adjusting) on a common effect of randomization and a variable typically leads to randomization being associated with the variable within strata, even if they were independent in the overall population.^3^

In this case, BMI is a plausible collider, as it is potentially causally downstream of inflammation and pre‐clinical dementia. Stratifying on BMI could therefore lead to *CRP* variants being associated with dementia within strata of BMI in the absence of a causal effect of inflammation. The pattern of genetic associations observed by Hegazy and colleagues is typical of that occurring due to collider bias: a null overall association, which is positive in one stratum and negative in the other.^4^ There is no clear biological reason that the direction of effect of CRP would be in the positive in low BMI individuals but in the inverse direction in high BMI individuals.

Generally speaking, adjustment for covariates other than age, sex, ancestry, and technical covariates (such as study center) is discouraged in Mendelian randomization analyses due to the possibility of inducing collider bias.^5^ Stratification on covariates is possible, but requires careful application of suitable methodology so as not to induce collider bias.^6^ In both randomized trials and Mendelian randomization, analysts and reviewers should be particularly skeptical of results from subgroup analyses where the overall association is null, and estimates for subgroup analyses are in opposing directions. Although there may be some cases where the direction of causal effect of a risk factor truly differs between subgroups, these are likely to be rare, and collider bias is often a more plausible explanation.

## CONFLICT OF INTEREST

None. Author disclosures are available in the supporting information.

## Supporting information



SUPPORTING INFORMATION

## References

[1] Hegazy SH , Thomassen JQ , Rasmussen IJ , Nordestgaard BG , Tybjærg‐Hansen A , Frikke‐Schmidt R . C‐reactive protein levels and risk of dementia – Observational and genetic studies of 111,242 individuals from the general population. Alzheimers Dementia. 2022;18(11):2262‐2271. 10.1002/alz.12568 PMC9790296 35112776

[2] Yusuf S , Wittes J , Probstfield J , Tyroler HA . Analysis and interpretation of treatment effects in subgroups of patients in randomized clinical trials. JAMA. 1991;266(1):93‐98. 2046134

[3] Cole SR , Platt RW , Schisterman EF , et al. Illustrating bias due to conditioning on a collider. Int J Epidemiol. 2010;39(2):417‐420. 19926667 10.1093/ije/dyp334 PMC2846442

[4] Gkatzionis A , Burgess S . Contextualizing selection bias in Mendelian randomization: how bad is it likely to be? Int J Epidemiol. 2018;48(3):691‐701. 10.1093/ije/dyy202 PMC6659463 30325422

[5] Burgess S , Davey Smith G , Davies NM , et al. Guidelines for performing Mendelian randomization investigations. Wellcome Open Res. 2019;4:186. 32760811 10.12688/wellcomeopenres.15555.1 PMC7384151

[6] Coscia C , Gill D , Benitez R , Perez T , Malats N , Burgess S . Avoiding bias in Mendelian randomization when stratifying on a collider. Eur J Epidemiol. 2022. In press. 10.1007/s10654-022-00879-0 PMC9329404 35639294

